# Assessment of the hepatic tumor extracellular matrix using elastin-specific molecular magnetic resonance imaging in an experimental rabbit cancer model

**DOI:** 10.1038/s41598-020-77624-8

**Published:** 2020-11-27

**Authors:** Sarah Keller, Tabea Borde, Julia Brangsch, Carolin Reimann, Avan Kader, Daniel Schulze, Rebecca Buchholz, Jan O. Kaufmann, Uwe Karst, Eyk Schellenberger, Bernd Hamm, Marcus R. Makowski

**Affiliations:** 1 Department of Radiology, Charité - Universitätsmedizin Berlin, corporate member of Freie Universität Berlin, Humboldt-Universität Zu Berlin, and Berlin Institute of Health, Charitéplatz 1, 10117 Berlin, Germany; 2 Department of Education Science and Psychology, FU Berlin, Berlin, Germany; 3 Institute of Inorganic and Analytical Chemistry, Westfälische Wilhelms-Universität Münster, Münster, Germany; 4 Division 1.5 Protein Analysis, Federal Institute for Materials Research and Testing (BAM), Berlin, Germany; 5 Department of Chemistry, Humboldt-Universität Zu Berlin, Berlin, Germany; 6 Department of Diagnostic and Interventional Radiology, School of Medicine & Klinikum Rechts Der Isar, Technical University of Munich, Munich (TUM), Munich, Germany

**Keywords:** Magnetic resonance imaging, Molecular imaging

## Abstract

To investigate the imaging performance of an elastin-specific molecular magnetic resonance imaging (MRI) probe with respect to the extracellular matrix (ECM) in an experimental hepatic cancer model. Twelve rabbits with hepatic VX2 tumors were examined using 3 T MRI 14, 21, and 28 days after tumor implantation for two subsequent days (gadobutrol, day 1; elastin-specific probe, day 2). The relative enhancement (RE) of segmented tumor regions (central and margin) and the peritumoral matrix was calculated using pre-contrast and delayed-phase T1w sequences. MRI measurements were correlated to histopathology and element-specific and spatially resolved mass spectrometry (MS). Mixed-model analysis was performed to assess the performance of the elastin-specific probe. In comparison to gadobutrol, the elastin probe showed significantly stronger RE, which was pronounced in the tumor margin (day 14–28: *P* ≤ 0.007). In addition, the elastin probe was superior in discriminating between tumor regions (χ^2^(4) = 65.87; *P* < 0.001). MRI-based measurements of the elastin probe significantly correlated with the ex vivo elastinstain (R = .84; *P* <0 .001) and absolute gadolinium concentrations (ICP-MS: R = .73, *P* <0 .01). LA-ICP-MS imaging confirmed the colocalization of the elastin-specific probe with elastic fibers. Elastin-specific molecular MRI is superior to non-specific gadolinium-based contrast agents in imaging the ECM of hepatic tumors and the peritumoral tissue.

## Introduction

The prevalence of hepatocellular carcinoma (HCC) is globally increasing with a high mortality rate^1^. Despite advances in therapeutic and diagnostic strategies in the last decades, the tumor resistance to treatment remains a major challenge for targeted therapy^2^. The tumor responsiveness to local and systemic therapy is not only dependent on the reduction of tumor cell proliferation, but also on the sensitivity of the peritumoral extracellular matrix (ECM) towards anticancer agents^3,4^ in various primary liver cancers and metastasis^5–7^. In the tumor microenvironment, stromal cells express an altered ECM that provides mechanical and biochemical protection to tumor cells and the surrounding microenvironment^2^. Collagens, laminins and fibronectins, in particular, directly and indirectly, interact with tumor cells and are able to change the function and phenotype due to external stimuli, such as therapy^8,9^. Elastin, another component of the ECM has recently been identified as an independent predictor of HCC development^10^. The VX2 rabbit model for liver cancer is a well-studied model for testing and evaluation of preclinical interventional^11,12^ and systemic therapies^13^ on an HCC surrogate tumor. A recent ultrasound-based therapy study has linked the importance of the ECM for therapy options by using microbubbles to influence the interstitial fluid pressure in this model^14^. Furthermore, it could already be shown that brachytherapy using the genetically engineered peptide polymer elastin-like polypeptide labelled with I(131) in the VX2 liver tumors has a strong labelling efficiency and thus a high antitumor effect^15^. Non-invasive magnetic resonance imaging (MRI) biomarkers could enhance the current knowledge about ECM interactions following tumor progression and therapy. However, no molecular contrast agents have yet been approved for the imaging of hepatic tumors in clinical routine.

Among the recently available standard extracellular MRI contrast agents, gadobutrol (gadolinium-DO3A-butriol, Gadovist 1.0; Bayer Schering Pharma, Berlin, Germany) is one of the first commercially available 1 M gadolinium chelates belonging to the class of macrocyclic, neutral gadolinium complexes^16–18^. Various comparative studies of 0.5 M and 1 M gadobutrol in experimental and clinical HCC have confirmed its effectiveness in tumor detection. In particular, gadobutrol 1 M shows high contrast efficiency and thus increased detectability even of small intrahepatic tumors^17,19^. In intramuscular VX2 rabbit tumors, 1 M gadobutrol showed increased contrast enhancement, better tumor-to-muscle differentiation and better delineation of the tumor border than 0.5 M gadopentatate dimeglumine^20^. However, with regard to the histopathological grading of HCC in patients, gadobutrol has not yet been shown to have a predictive effect^21^. Furthermore, there are no studies that have investigated whether gadobutrol can differentiate the different tumor regions and their ECM. With reference to the above, these data are of particular interest for a better assessment of the therapeutic effects of invasive and systemic procedures.

Molecular elastin-specific MR contrast agents have been already successfully used to study the ECM remodeling in cardiovascular disease with results indicating a large translational potential^22–24^. In this context, elastin-specific molecular MRI could not only quantify the elastin content in arteriosclerotic plaques on the basis of signal intensity, but also predict potential rupture sites in the course of an aortic aneurysm in follow-up studies^22,23^. In a mouse model for Marfan's disease, elastin-specific MRI reliably detected a decrease in aortic wall elastin concentration compared to wild-type controls^25^. To date, there are no records of studies that have used elastin-specific molecular MRI to characterize the ECM in hepatic tumors. Assuming that hepatic tumors have an increased expression of collagens, including elastin^10^, the hypothesis arises that elastin-specific contrast agents allow for a more clearly defined enhancement and differentiation of tumor regions based on the composition of their ECM than conventional gadolinium-based contrast agents.

The aim of this study was to test the potential of an elastin-specific molecular MR probe to assess and quantify the peritumoral matrix in a rabbit VX2 hepatic cancer model.

## Results

### MR imaging

**Figure 1:**
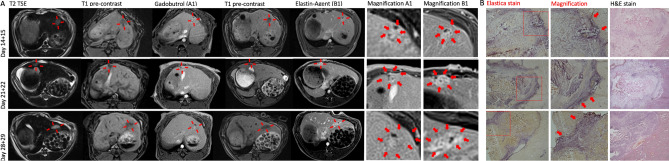
In vivo and ex vivo imaging of untreated VX2 liver tumors. (**A**) MR imaging of the liver at three different time points (day 14 + 15, 21 + 22, 28 + 29) following implantation. Left–right: T2w imaging, unenhanced T1 weighted imaging (day 1), gadobutrol enhanced T1 weighted imaging (day 1, A1), unenhanced T1 weighted imaging (day 2), elastin-specific MR probe T1 weighted imaging (day 2, B1), magnification of A1 and B1. (**B**) Histopathology using Elastica van Giesson and H&E stain. Strong expression of elastic fibers in the hepatic tumor, pronounced in the tumor margin. 2 × magnification (Elastica and H&E stain), 10 × magnification (Elastica stain magnification).

The average tumor volume measured in the native T1 sequences was 0.41 (±0.38) cm^3^. After intravenous contrast agent administration of gadobutrol on day 1, there was a shallow enhancement of the tumor regions, accentuated during the venous and late contrast agent phase. Tumor enhancement was visually potentiated after administration of the elastin-specific probe on day 2 (Fig. 1A). The increased enhancement using the elastin-specific probe corresponded to the ex vivo histological EvG stain (Fig. 1B). The pronounced enhancement following the injection of the elastin-specific probe was confirmed by the quantitative MR image analysis.

### Relative enhancement (RE)

**Figure 2:**
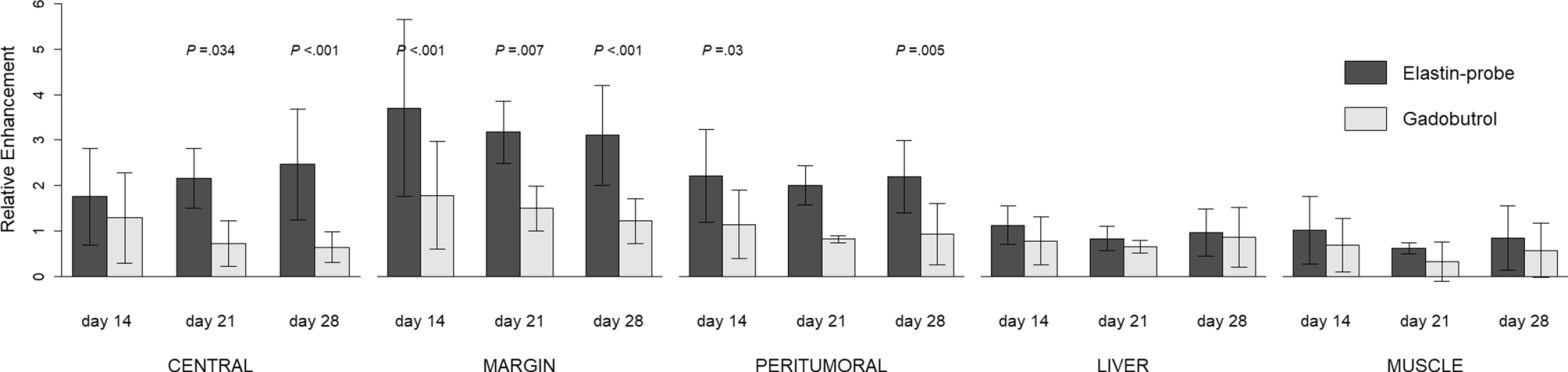
Relative Enhancement (RE) depending on the contrast agent and latency time for the different regions. RE for the liver and muscle is included as reference. Error bars represent standard deviation. P values indicate significant differences between the two agents. R^36^ (Version 1.2.5001, R-Development-Core-Team, 2019) was used to create the graphics.

Time and region had a significant impact on RE when compared to the null model (*χ*^2^(14) = 69.23, *P* < 0.001). More importantly, the applied contrast agent had an additional significant effect above time and region (*χ*^2^(15) = 120.83, *P* < 0.001). The elastin-specific contrast agent showed a significant higher RE compared to gadobutrol in the central, marginal, and peritumoral region. This difference was pronounced in the tumor margin (Fig. 2). Post hoc tests were carried out to gain further insight into the differences of the agents depending on regions and time points. In the post hoc analysis, tumor areas showed significant differences between gadobutrol and elastin-specific contrast uptake in the tumor margin on day 14 (difference 1.86; *P* < 0.001), day 21 (difference 1.68; *P* = 0.007), and day 28 (difference 1.81; *P* < 0.001). In the peritumoral region, gains of elastin-specific probes were observed on day 14 (difference 1.01; *P* = 0.03) and day 28 (difference 1.18; *P* = 0.005), in the central regions on day 21 (difference 1.44; *P* = 0.034) and day 28 (difference 1.75; *P* < 0.001) (Table 1). The RE difference between the two agents in normal liver parenchyma and back muscles was not significant at all time points (*P* = 1.00). Taken together for all time points, the RE using the elastin-specific probe differed significantly between the tumor regions (χ^2^(4) = 65.87, *P* < 0.001). In detail, there were significant differences in the RE of the elastin-specific probe between the tumor center and margin (difference − 1.24; *P* < 0.001), the tumor margin and the peritumoral region (difference 1.20, *P* < 0.001) and the peritumoral region and the liver parenchyma (difference 1.16, *P* < 0.001) (Table 2). In contrast, the RE of gadobutrol was more unspecific and only distinguished between central and margin (difference − 0.59; *P* < 0.05) and between margin and liver (difference 0.71; *P* < 0.01). The difference in RE between margin and peritumoral tissue and between peritumoral and liver parenchyma was not significant (difference 0.51; *P* = 0.065 and difference 0.20; *P* = 1.00, respectively).

**Table 1:** Differences in relative enhancement (RE) between the enhancement of the elastin-specific probe and gadobutrol.

Time	Region	CA difference	*t*	*P*
14 days	Central	.40	1.30	1.00
	Margin	**1.86**	6.01	**< .001**
	Peritumoral	**1.01**	3.25	**.030**
	Liver	.28	.91	1.00
	Muscle	.26	.826	1.00
21 days	Central	**1.44**	3.23	**.034**
	Margin	**1.68**	3.67	**.007**
	Peritumoral	1.18	3.77	.168
	Liver	.18	.41	1.00
	Muscle	.29	.66	1.00
28 days	Central	**1.75**	5.67	**< .001**
	Margin	**1.81**	5.85	**< .001**
	Peritumoral	**1.18**	3.83	**.005**
	Liver	.03	.09	1.00
	Muscle	.19	.60	1.00

Positive difference values indicate an advantage of the elastin-specific probe.

CA contrast agent.

Bold print: significant differences (*P* ≤ .05).

**Table 2:** Differentiability of the regions based on the relative enhancement (RE) after application of gadobutrol (upper right section) and the elastin-specific probe (lower left section) together for all examination time points.

	Gadobutrol				
	Central	Margin	Peritumoral	Liver	Muscle
Central	1.52***	− .59*	− .08	.13	.35
Margin	− 1.24***	2.43***	.51	.71**	.93**
Peritumoral	− .04	1.20***	1.58***	.20	.42
Liver	1.12***	2.36***	1.16***	.90***	.22
Muscle	1.26***	2.50***	1.30***	− .14	.72**
	Elastin-specific probe				

Diagonal (bold print): strength of region discrimination (Bonferroni-corrected) using the elastin-specific probe compared to gadobutrol. Upper section: crude differences in gadobutrol RE between regions (central, margin, peritumoral, liver, muscle). Lower section: crude differences in elastin-specific RE between regions (central, margin, peritumoral, liver, muscle).

*P < .05; **P < .01; ***P < .001.

### Histopathology

**Figure 3:**
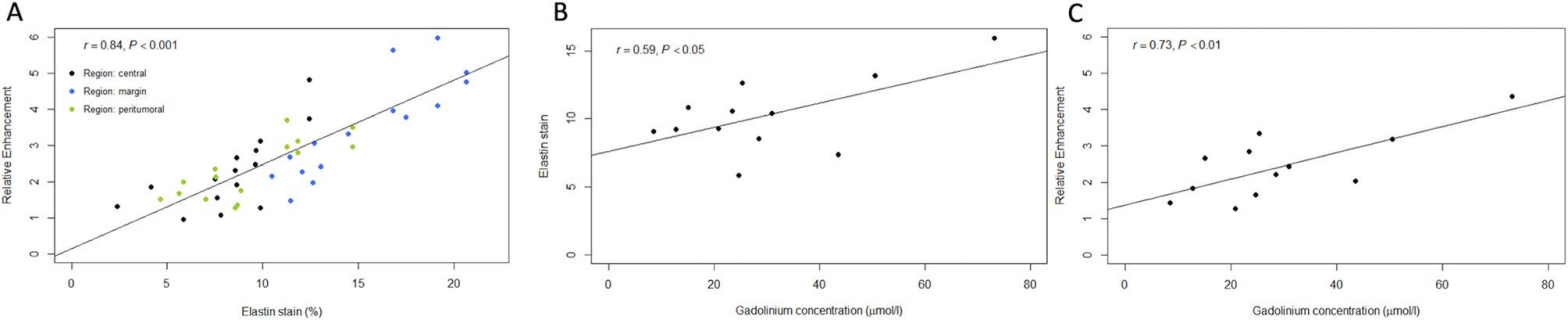
Associations of in vivo MR RE and ex vivo histological and mass spectrometry quantification of elastin in the tumor regions. (**A**) Correlation of in vivo RE and ex vivo quantification of the elastin stain area on cryosections. The RE of elastin probe measurements significantly correlates (R = 0.84, *P* < 0.001) with the histopathological expression of elastic fibers (elastin stain) in the three tumor regions. (**B**) Linear regression shows a significant correlation between the elastin stain and the ICP-MS gadolinium concentration (µmol/l) of the whole tumor (R = 0.59; *P* < 0.05). (**C**) Linear regression shows a significant correlation of the RE and gadolinium concentration (µmol/l) (R = 0.73; *P* < 0.01). This indicates a close association between the amount of in vivo binding of the elastin-specific molecular probe and the overall gadolinium detected in tissue. R^36^ (Version 1.2.5001, R-Development-Core-Team, 2019) was used to create the graphics.

Viable tumors were found in all animals. Tumor regions were visualized on H&E and EvG stained sections and corresponded to the respective slight on the acquired MR images (Fig. 1). The in vivo RE measurements of the three tumor areas correlated significantly to the ex vivo EvG stain area measurements (R = 0.84, *P* < 0.001) (Fig. 3A).

### ICP-MS

The average concentration of gadolinium in the whole tumor was quantified using ICP-MS. The ICP-MS gadolinium concentration (µmol/l) of the whole tumor correlated significantly to histopathological elastin area stains (%) (R = 0.59; *P* < 0.05) and the elastin-specific MR-based RE (R = 0.73; *P* < 0.01) (Fig. 3B,C).

### LA-ICP-MS

**Figure 4:**
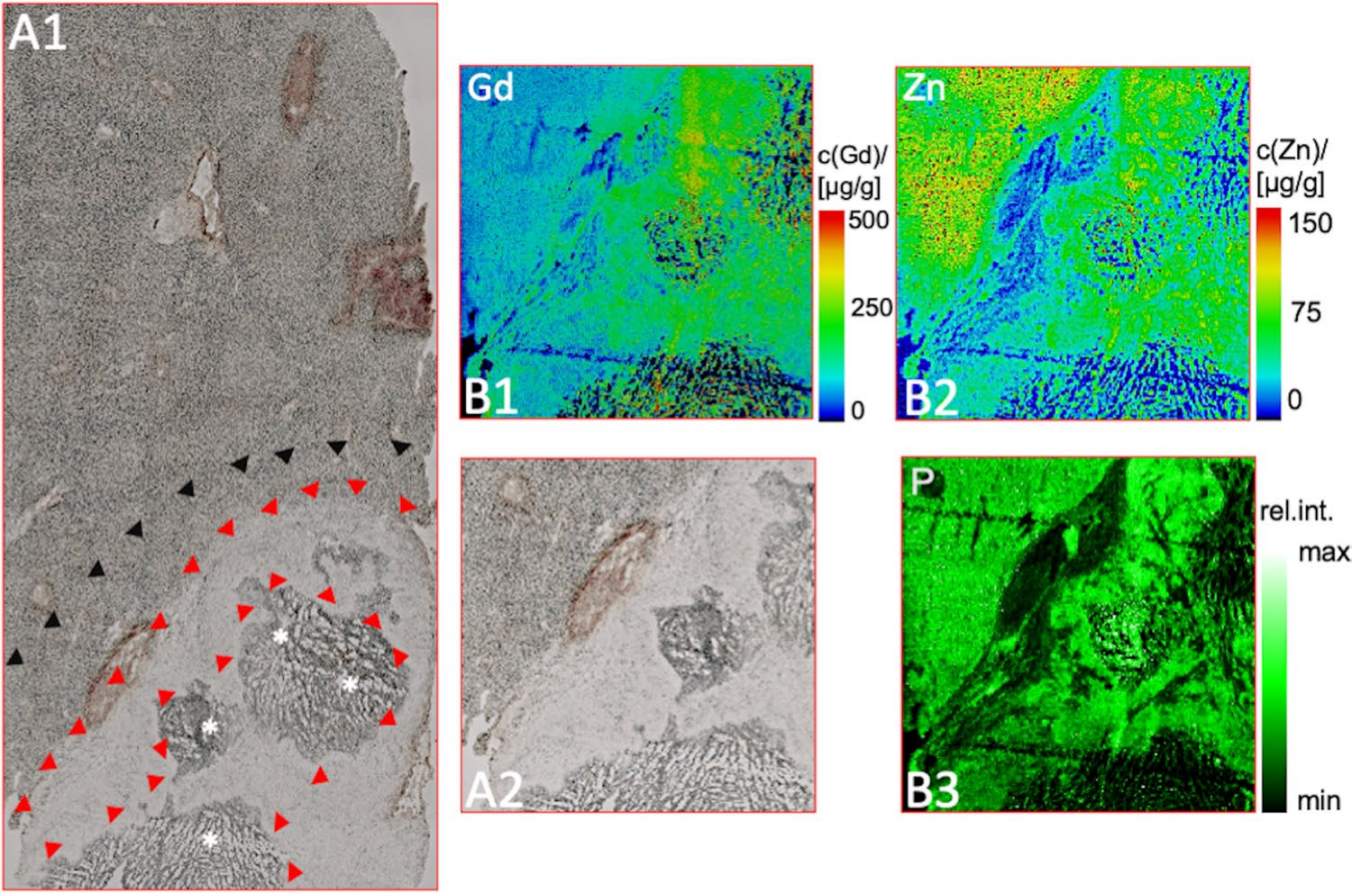
LA-ICP-MS for the assessment of the gadolinium distribution in the tumor areas. (**A1**, **A2**) Unstained cryosection for anatomical matching of the tumor (**A2** magnification). The arrowheads mark the tumor margin (red) and the peritumoral region (black). The asterisks (*) highlight the central zone. (**B1**) Specific gadolinium distribution of the magnified area (A2) confirms gadolinium in all tumor regions, predominantly in the tumor margin (green signal). (**B2**, **B3**) Control measurements regarding zinc (Zn) and phosphorous (P) do not show a specific distribution inside the tumor.

The gadolinium distribution was determined by LA-ICP-MS within the three regions and the surrounding liver parenchyma following the in vivo elastin-specific MRI on the second day. The colocalization of the gadolinium-bound elastin-specific probe with elastic fibers was pronounced in the tumor margin and the peritumoral region and corresponded to increased zinc distribution. The spatial distribution of P, Zn and Gd in the samples were not specifically distributed within the samples (Fig. 4).

## Discussion

The major finding of this study is that molecular MR imaging using the elastin-specific probe enables the discrimination of the different tumor regions and the peritumoral matrix with a higher accuracy compared to conventional gadolinium-based contrast agents in VX2 hepatic tumors. Mass spectroscopic and histopathological quantification of elastin accurately confirmed imaging-derived increases in elastin-specific contrast enhancement on MRI especially in the tumor margins. The concise differentiation of the tumor center, margin and immediate peritumoral space addresses an unmet clinical need for a non-invasive imaging protocol to detect metabolic alterations and assess therapy responsiveness. The imaging findings of the current study have been correlated to different modalities of ex vivo MS- and histopathology-based quantification techniques, thus providing a ground truth validation for the in vivo measurements. Therefore, this finding may improve the standard imaging protocol of treatment studies in experimentally induced hepatic cancer models in the future and potentially in patients.

Three tumor-associated regions were differentiated to address the variability of cells and ECM and thus the varying responsiveness to therapeutic agents and interventional studies. Whereas the central tumor region is mainly composed of necrotic and inactive cells, viable cells within the tumor margin contribute to tumor growth and progression. These cells interact within an elastic extracellular microenvironment, that provides not only chemical signals but also physical stimuli through stiffness^26,27^. The peritumoral matrix including fibroblasts, inflammatory cells, lymphatic vascular networks and different collagens as well as elastic fibers, has long been thought of as a stromal barrier to contain tumor progression by regulating various anti-cancer pathways. However, it has been shown that once transformed into a tumor-associated neighborhood by various stimuli, the stromal-derived effects can actively contribute to the tumor aggressiveness and responsiveness to anti-tumor therapies^2,28^. In a recently published US- based study the application of US microbubbles of different intensity and the response of the interstitial fluid pressure in the ECM was investigated^14^. It can be anticipated that more therapeutic studies will focus on the ECM tumor component in the future.

Few studies aimed to prove the linkage between the elastic ECM and peritumoral matrix, tumorigenesis and tumor progression. Yasui et al*.*^10^ confirmed the predictive role of increased elastin fiber accumulation within the hepatic ECM for the development of HCC in patients. Maehara et al*.*^29^ examined histopathological specimens of explanted human HCC and observed increased elastin and elastin-collagen components within the tumors. Moreover, increased elastin levels were associated with increased inflammatory cell infiltration, smaller capsule formation, and higher percentage of scirrhous stroma^29^. Their ratio of elastin to collagen was significantly higher in the tumor fibrous capsule (*P* < 0.007). However, whereas iodine-based contrast-enhanced CT correlated to the tumor collagen content, it failed to specifically detect elastic fibers. The finding of Maehara et al*.*^29^ corresponds to our study results that conventional GBCAs are inferior to the elastin-specific MR agent in terms of elastin in tissue. Non-invasive MR imaging using the elastin-specific molecular probe produced image data with a high spatial resolution and clear contrast enhancement in the viable tumor regions, thus underlining the priority of targeted MRI in hepatic cancer research. Especially in the area of targeted therapies of tumor associated ECM components or cancer to stroma signaling cross-talks, such as sorafenib and erlotinib, elastin-specific molecular imaging could act as a potential imaging biomarker to demonstrate the tumor responsiveness to the therapeutic agent.

Some limitations of this study have to be acknowledged. Firstly, the study cohort consisting of N = 9 cross-sectional and N = 3 longitudinal rabbits was relatively small but comparable to previously published research in this area^30–32^. Secondly, the MRI was performed in free breathing, but under deep sedation which considerably reduces breathing motion. Thirdly, even though we observed a clear pattern of elastin-specific contrast agent accumulation within the tumor regions, a longitudinal effect on the specific enhancement patterns was not observed, indicating that tumor remodeling with regards to elastic fibers does not seem to take place in untreated settings within the first four weeks following tumor implantation. The missing tumor remodeling could be used on the other hand to use the available data as a baseline imaging component for longitudinal studies with longer time frames.

In conclusion, our data confirm the diagnostic potential of the elastin-specific targeted MRI for imaging and quantitative assessment of the intra- and peritumoral matrix in a hepatic cancer animal model. Molecular MRI using the elastin-specific probe enables the discrimination of the different tumor regions and the peritumoral matrix with a higher accuracy compared to conventional gadolinium-based contrast agents in VX2 hepatic tumors. This technique might be useful to assess systemic and local treatment effects in future experimental and clinical studies.

## Materials and methods

### Animal model

All experimental protocols were performed according to the guidelines and regulations of the Federation of Laboratory Animal Science Associations (FELASA) and the local Guidelines and Provisions for Implementation of the Animal Welfare Act, as approved by the Regional Office for Health and Social Affairs Berlin (LAGeSo) (registration number 0178/17). Twelve female New Zealand white rabbits (Charles River Laboratories, Sulzfeld, Germany) aged 11–17 weeks, mean weight (standard deviation) 3.2 ± 0.3 kg were used for this experimental study. VX2 cells were injected into the hindlimb muscles of six female donor rabbits and grown for 21–30 days to a size of 1.5–2.0 cm as previously described^33^. Harvested chunks were minced and implanted into the left liver lobe by mini laparotomy into the receiver rabbits (N = 12). Anesthesia was performed using intravenous injections of Buprenorphin (Temgesic, 0.03 mg/kg body weight) and subcutaneous injections of medetomidine hydrochloride (Cepetor, 0.25 mg/kg body weight), and ketamine hydrochloride (Ketamin, 30 mg/kg body weight). Carprofen (Rimadyl, 4.0 mg/kg body weight) was injected as an analgesic for the following three days after all surgical procedures.

**Figure 5:**
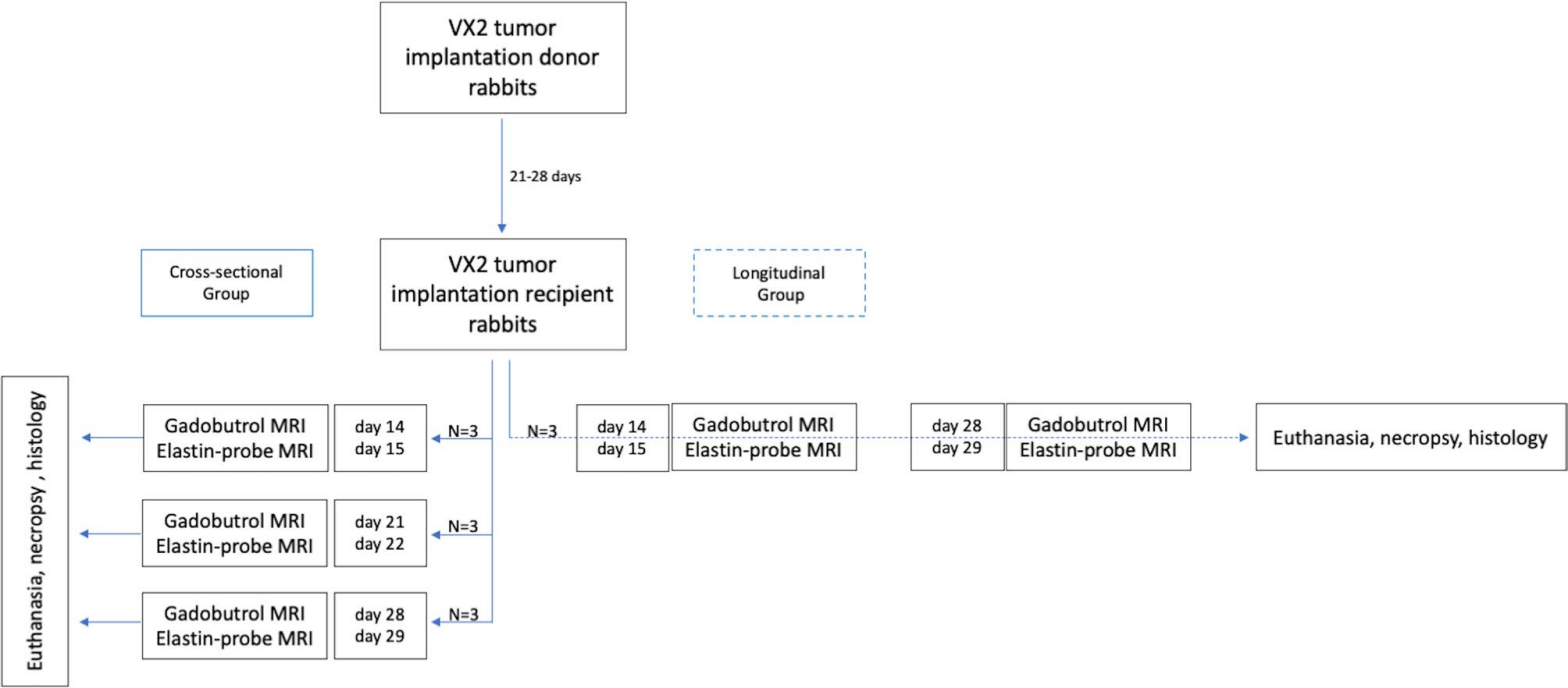
Experimental in vivo study design. In the vertical direction, the flow chart illustrates the VX2 rabbit tumor model and multimodal imaging on sequential time points. Left column: groups of three tumor-bearing rabbits were assigned to one MRI time point (day 14 + 15, 21 + 22, and 28 + 29 after tumor implantation) and euthanized following the scan. Right column: a group of three rabbits was assigned to two longitudinal MRI scans at each time point (14 + 15 and 28 + 29 days) and euthanized following the scan at time point 29.

According to the study protocol (Fig. 5), the rabbits were randomized into four groups following tumor implantation. Three cross-sectional groups with N = 3 rabbits each received an MRI scan 14 + 15, 21 + 22, or 28 + 29 days after tumor implantation immediately followed by euthanasia using intravenous injection of pentobarbital sodium (Narcoren, 300 mg/kg body weight), and necropsy. A longitudinal group with N = 3 tumor bearing rabbits was additionally imaged 14 + 15 and 28 + 29 days after tumor implantation and euthanized on day 29.

### Gadolinium-based elastin probe

The elastin-specific molecular agent (ESMA; Lantheus Medical Imaging, North Billerica, MA) is composed of the D-amino-acid homophenylalanine, which is coupled by a rigid hydrazine aminomethylbenzoic acid linker to a gadolinium-diethylenetriaminepentaacetic acid complex^22^. Ex vivo measurements obtained a longitudinal relaxivity for the agent bound to mice aortas of 8.65 ± 0.42 mmol/l^−1^ s^−1^ at 3 T^22^. In this study, a clinical dose of 0.2 mmol/kg was used and administered as a bolus via the ear vein.

### MR imaging

MRI was performed under deep sedation using a 3 T clinical scanner (mMR Biograph, Siemens Medical Solutions, Erlangen, Germany). The rabbits were imaged in prone position using a clinically approved head-neck coil. Each rabbit was scanned on two consecutive days. Animals received an intravenous injection of gadobutrol (Gadovist 1.0 mmol/ml, Bayer Healthcare AG, Berlin) 0.2 mmol/kg on the first day. On the second day an elastin-specific gadolinium-based probe was administered at 0.2 mmol/kg. Anatomic images were acquired using a T2-weighted (TR/TE 5500/90 ms, FOV 180 × 180 mm^2^, voxel size 0.5 x 0.5 × 1.0 mm^3^, NSA 3) and a clinically approved three-dimensional (3D) gradient-recalled-echo (GRE) sequence, which is routinely applied as a standard sequence for liver imaging and liver tumor detection^34^ (T1 volumetric interpolated breath-hold examination (VIBE), TR/TE 5.0/2.18 ms, FOV 180 × 180 mm^2^, voxel size 0.7 × 0.7 × 1.0 mm^3^, NSA 2), before and 20 min after injection of the contrast agent. The T1 VIBE sequence allows improved MR imaging by providing dynamic contrast–enhanced thin-section images with fat saturation and a high signal-to-noise ratio^35^. Additional pre-contrast scans were performed for each animal on the second day prior to injection of the elastin-specific probe to detect any residual retention of gadobutrol in the liver of the animals.

### Image postprocessing

MR images were analyzed by two radiologists in consensus using HOROS (v 4.0.0.0RC1; Nimble Co LLC d/b/a Purview in Annapolis, MD USA; https://horosproject.org). Tumors were manually segmented into a central and marginal region. Additional regions of interest (ROIs) covered the peritumoral matrix, normal liver parenchyma and the back muscles of the same slight. The central area also covered the tumor necrosis, the marginal area included microscopically densely packed the “vital” tumor cells and the tumor fibrous capsule.

The relative enhancement (RE) was assessed using the following formula:

1 $$RE = \frac{{(SI_{postcontrast} - SI_{precontrast} )}}{{SI_{precontrast} }}.$$

### Histology

Following the MRI scan, liver tumors were explanted and immediately frozen at − 80 °C. 10 µm cryosections mounted on adhesion slides (SuperFrost Plus, Thermo Scientific) were stained with Miller’s elastic van Gieson histochemical stain (EvG) and hematoxylin and eosin stain (H&E) to visualize the tumor, ECM and elastic fibers. A light microscope (BzX800, Keyence, Japan) was used for examination of the slides. Digitalized images (TIFF file format) were stored for computer-assisted image analysis (ImageJ software, version 1.51, Wayne Rasband, National Institutes of Health; https://imagej.nih.gov/ij/). To measure the %EvG stain area per region all structures within the specific color profile were automatically segmented. By dividing the segmented area by the respective region (e.g. peritumoral), the %EvG stain area was determined.

### Laser ablation-inductively coupled plasma-mass spectrometry (LA-ICP-MS) for elemental bioimaging

The LA-ICP-MS analysis for quantitative imaging of gadolinium (Gd), iron (Fe), zinc (Zn), and phosphor (P) was conducted as previously described^24^. Due to the different sample composition, a scan speed of 45 µm/s with unchanged 800 ml/min helium (He) as transport gas was used in this analysis. In this analysis the averaged intensities of the scanned lines of the standards demonstrated a linear correlation with a R^2^ = 0.996 over the concentration range. The limit of detection (LOD) and limit of quantification (LOQ) were calculated using 3σ- and 10σ-criteria and were as follows: 12 ng/g and 40 ng/g for Gd, 5.9 µg/g and 20 µg/g for Fe and 2.0 µg/g and 6.6 µg/g for Zn, respectively.

### Inductively coupled plasma mass spectroscopy (ICP-MS)

For the ICP-MS analysis, samples were digested in 70% nitric acid at 37 °C overnight immediately after the last imaging session, followed by dilution with deionized water for ICP-MS analysis. A standard curve was acquired with each sample set for gadolinium concentration determination.

### Statistical analysis

The statistical software ‘R’^36^ (Version 1.2.5001, R-Development-Core-Team, 2019) with the package ImerTest providing mixed model methods^37^ was used for statistical analysis. First, we used analyses of variance (ANOVAs) for each time point separately to analyze the cross-sectional part of the data. Additionally, we applied Bonferroni corrected t-tests to compare the differences between both agents for all regions and time points. Second, we applied a mixed model to account for the cross-sectional and longitudinal combined study design with planned missings. RE was the dependent variable. Time latency (14, 21, or 28 days), region (central, margin, peritumoral, liver, and muscle), and the agent (gadobutrol or elastin-specific probe), as well as their interactions, were the independent variables. To display the overall effect of a variable, we used likelihood ratio tests; the effects of specific variable stages were assessed with *t*-tests following Satterthwaite’s method^38^.

### Ethics approval and consent to participate

According to ARRIVE guidelines this study was approved by the local guidelines and provisions for the implementation of the Animal Welfare Act and regulations of the Federation of Laboratory Animal Science Associations (FELASA; registration number 0178/17).

## Data Availability

The datasets used and/or analyzed during the current study are available from the corresponding author on reasonable request.

## Abbreviations

ECM: Extracellular matrix
ESMA: Elastin-specific molecular agent
EvG: Miller’s elastic van Gieson
FELASA: Federation of Laboratory Animal Science Associations
GRE: Gradient-recalled-echo (sequence)
H&E: Hematoxylin and eosin
HCC: Hepatocellular carcinoma
ICP-MS: Inductively coupled plasma mass spectroscopy
LA-ICP-MS: Laser ablation-inductively coupled plasma-mass spectrometry
MRI: Magnetic resonance imaging
RE: Relative enhancement
US: Ultrasound
VIBE: Volumetric interpolated breath-hold examination
